# Thermo-neutrophilic cellulases and chitinases characterized from a novel putative antifungal biocontrol agent: *Bacillus subtilis* TD11

**DOI:** 10.1371/journal.pone.0281102

**Published:** 2023-01-27

**Authors:** Muhammad Saqib Malik, Abdul Rehman, Irfan Ullah Khan, Taj Ali Khan, Muhammad Jamil, Eui Shik Rha, Muhammad Anees

**Affiliations:** 1 Department of Microbiology, Kohat University of Science and Technology, Kohat, Pakistan; 2 Vaccine Development Group, Animal Sciences Division, Nuclear Institute for Agriculture and Biology, Faisalabad, Pakistan; 3 Department of Microbiology, Khyber Medical University Peshawar, Khyber Pakhtunkhwa, Pakistan; 4 Department of Biotechnology and Genetic Engineering, Kohat University of Science and Technology, Kohat, Pakistan; 5 Department of Well-Being Resources, Sunchon National University, Suncheon, Republic of Korea; Benemérita Universidad Autónoma de Puebla: Benemerita Universidad Autonoma de Puebla, MEXICO

## Abstract

Cellulose and chitin are the most abundant naturally occurring biopolymers synthesized in plants and animals and are used for synthesis of different organic compounds and acids in the industry. Therefore, cellulases and chitinases are important for their multiple uses in industry and biotechnology. Moreover, chitinases have a role in the biological control of phytopathogens. A bacterial strain *Bacillus subtilis* TD11 was previously isolated and characterized as a putative biocontrol agent owing to its significant antifungal potential. In this study, cellulase and chitinase produced by the strain *B*. *subtilis* TD11 were purified and characterized. The activity of the cellulases and chitinases were optimized at different pH (2 to 10) and temperatures (20 to 90°C). The substrate specificity of cellulases was evaluated using different substances including carboxymethyl cellulose (CMC), hydroxyethyl cellulose (HEC), and crystalline substrates. The cellulase produced by *B*. *subtilis* TD11 had a molecular mass of 45 kDa while that of chitinase was 55 kDa. The optimal activities of the enzymes were found at neutral pH (6.0 to 7.0). The optimum temperature for the purified cellulases was in the range of 50 to 70°C while, purified chitinases were optimally active at 50°C. The highest substrate specificity of the purified cellulase was found for CMC (100%) followed by HEC (>50% activity) while no hydrolysis was observed against the crystalline substrates. Moreover, it was observed that the purified chitinase was inhibitory against the fungi containing chitin in their hyphal walls i.e., *Rhizoctonia*, *Colletotrichum*, *Aspergillus* and *Fusarium* having a dose-effect relationship.

## Introduction

Biological control of plant disease caused by a variety of pathogenic organisms is a well-known technique used now-a-days. It is an economically friendly alternative to synthetic pesticides. The conventional practices have adverse impacts not only on the soil fertility but also on ecological and terrestrial environment [1]. Biological control agents (BCA) are mostly soil-borne microorganisms that may play a vital role in the eradication of phytopathogens [2]. Different bacterial species have been reported as BCA and among them *Bacillus* and *Pseudomonas* are the most common bacterial genera found in the rhizospheric regions of plants [3, 4]. It has also been reported that the species of *Bacillus* not only protect plants from different phytopathogens but may also promote their growth by different mechanisms including provision of nutrients, secretion of growth stimulating compounds, mycoparasitism, antibiosis, and production of secondary compounds such as lytic enzymes that induce systemic resistance to fungal pathogens [5, 6]. Sagar et al. [7] have recently reviewed in detail and reported that different strains of *Bacillus* spp. not only show antifungal mechanisms against a variety of phytopathogenic fungi but also have versatile plant growth promoting properties.

The lytic enzymes isolated from bacteria are also important having wide variety of applications in textile, beverages, and pharmaceutical industries because of their catalytic activity to convert complex compounds into useful by-products [8]. Different microbial species such as *B*. *subtilis* [9], *Penicillium* [10], *Aspergillus* [11] and *Thermomonospora* [12] have been reported to produce cellulase and chitinase enzymes. Cellulose is the most abundant polysaccharide and the most predominant renewable agricultural waste in the world [13]. It can be catalyzed with the help of cellulase enzyme to glucose and then converted to different organic compounds. More than 20% of synthetic cellulolytic enzymes are available in the market, however, the bacteria-based cellulases are the most efficient, cost effective, and environmentally friendly having no toxicity to terrestrial life [14]. In addition to this, different mechanisms such as mycoparasitism, antibiosis, and production of lytic enzymes are involved in the biological control of fungi and fungi like organisms [15]. Among different extracellular lytic enzymes produced by microorganisms, cellulase is considered an important enzyme used to degrade cellulose, which is an integral component of hyphal walls of different phytopathogenic oomycetes [16].

Chitin is also among the most abundant polymer on the earth and can be hydrolyzed to various products. The chitinases have potential to hydrolyze the insoluble chitin to its different constituents i.e., n-acetyl glucosamine and other monomeric units, present in the cell wall of fungi [17]. Different bacterial species produce chitinases, and among them the well-known genera comprise of *Bacillus*, *Actinomycetes* and *Streptomyces* spp. Moreover, in the pharmaceutical industry, chitinase has wide range of applications i.e., used for the preparation of important antifungal agents, preparation of potential proteins, treatment of extracellular waste and control of malarial parasite [18].

In the present study, standard extraction and purification techniques were used for the characterization of cellulase and chitinase enzymes produced by a potent antagonistic strain of *B*. *subtilis* TD11, which was previously isolated and identified as a putative biocontrol agent owing to its versatile mechanisms against the plant pathogenic fungi including production of lytic enzymes [19]. Further, the activity of cellulase and chitinase enzymes was optimized in the present study at different pH (2 to 10) and temperature conditions (20–90°C). The substrate specificity of cellulases was also evaluated using carboxymethyl cellulose (CMC), hydroxyethyl cellulose (HEC). The purified chitinase enzyme was also used in bioassays against the phytopathogenic fungi.

## Materials and methods

### Microorganism

The antagonistic strain of *Bacillus subtilis* TD11 was previously isolated from rhizospheric soil and characterized for antagonistic activity against the phytopathogenic fungi [19]. The bacterial culture was maintained on nutrient agar and Luria Bertani (LB) agar plates.

### Preparation of Bacterial Culture Filtrate (BCF)

The bacterial strain (*B*. *subtilis* TD11) was grown overnight in 250 ml flask containing media (KH_2_PO_4_ 0.5 g, glucose 0.5 g, FeSO_4_ 0.01 g, peptone 0.75 g, MgSO_4_ 0.5 g in one litre). The culture medium was then incubated in shaking incubator at 170 rpm and 37°C for 5 days (New Brunswick Scientific, USA). After incubation, the culture was centrifuged (High speed refrigerated centrifuge, Esco-Versati, T1000R, Singapore) to remove the cellular debris. The resultant supernatant was then filtered out through a 0.2 μm filter paper (Whatman, UK). This cell free culture filtrate was then used for enzyme activity, extraction, and purification assays.

### Assays for lytic enzyme activity by *B*. *subtilis* TD11

#### Cellulase activity assay

To determine the cellulase activity at 50°C, 100 μl of appropriate concentrations of enzyme solutions were mixed with 1% carboxymethyl cellulose (CMC) in 20 m-mol phosphate buffer (pH 7.0) and incubated for 5 days. The activity was measured after every 24 hr. Instead of pure enzyme, 1 ml of distilled water was run along with the test enzyme as a control. Dinitrosalicylic acid (DNS) reagent was added to stop the reaction. The final solution was then cooled, and the absorbance was measured at 540 nm. One unit of enzyme activity was defined as the amount of enzyme that could hydrolyse CMC and release 1 μg of glucose within 1 min reaction at 50°C [20]. The data were analysed using one-way analysis of variance (ANOVA) by software ‘Statistix 9’ and the means were compared by using Fisher’s least significant difference (LSD) test.

#### Gelatinase activity assay

The enzyme solution (0.05 ml) was taken in an Eppendorf tube containing 1.0 ml of Tris-HCl buffer (pH 8) and 0.5% gelatine. The mixture was incubated at 37°C for 5 days. The activity was measured after every 24 hr. The enzyme reaction was stopped by adding 6 ml of pure trichloroacetic acid and was kept at refrigerator for 15 min. The mixture was then centrifuged in refrigerated centrifuge machine for about 5 min at 10,000 g. The supernatant (0.05 ml) was vortexed with 0.5 ml of 1–3% Ninhydrin solution and boiled for 20 min. After dilution with 50% n-propanol, the absorbance of the reaction mixture was measured at 570 nm [21]. The data were analysed using one-way ANOVA by software ‘Statistix 9’ and the means were compared by using LSD test.

#### Pectinase activity assay

The enzyme extract (1 ml) was added in a solution containing 2 ml of 1% pectin along with 0.2 M sodium-acetic acid buffer at pH 5.0 [21]. The mixture was incubated at 45°C for 5 days. The activity was measured after every 24 hr. One unit of pectinase activity was defined as the amount of enzyme which catalysed the substrate and produced 1 μg of galacturonic acid per hour at 45°C. Enzyme activity was measured in 1 μl ml^-1^min^-1^ by dividing the product of absorbance and standard factor by time of incubation. The data were analysed using one-way ANOVA by software ‘Statistix 9’ and the means were compared by using LSD test.

#### Protease activity assay

To find out the protease activity of *B*. *subtilis* strain TD11, 1 ml of filtrate solution was added in a mixture containing 0.5 ml of skimmed milk as the substrate and incubated at room temperature for 5 days. The activity was measured after every 24 hr. About 5 mL of 110 m-mol-L^-1^trichloroacetic acid (TCA) was then added to stop the enzyme reaction. The supernatant was collected by centrifugation and added to 5 ml of alkaline solution. The mixture was then incubated at room temperature for 30 min. The protease activity was measured by taking absorption peak at 650 nm in a UV-Vis spectrophotometer [22]. The data were analysed using one-way ANOVA by software ‘Statistix 9’ and the means were compared by using LSD test.

#### Chitinase activity assay

About 0.5 ml of the enzyme solution was added in a mixture containing 0.45 ml sodium acetate buffer (pH 5.0) and 0.5 ml of 0.5% colloidal chitin. About 0.2 ml of 1 mol sodium hydroxide solution was then added in the mixture and incubated at 37°C for 5 days. The activity was measured after every 24 hr. The mixture was subjected to heating for 15 min in boiling water to stop the reaction. Then 0.75 ml of supernatant was collected by centrifugation at 10,000 g for 5 min at 4°C and mixed with Schale’s reagent [21]. After this, the chitinase activity was measured by taking absorption peak at 420 nm using a UV-Vis spectrophotometer. The data were analysed using one-way ANOVA by software ‘Statistix 9’ and the means were compared by using LSD test.

### Extraction and purification of cellulase enzyme

#### Anion exchange chromatography

The fermented broth was centrifuged at 15,000 rpm for 20 min and the supernatant was passed through a 0.2 μm filter paper (Whatman, UK), and 80% saturated ammonium sulphate was used to precipitate the crude cellulase. The saturated solution was left overnight at 4°C and then centrifuged at 10,000 g for 20 min to get the precipitate. The precipitate was dissolved in a small volume of 0.05 mol sodium acetate buffer solution (pH 8.0) and left overnight in dialysis membrane.

The DEAE-cellulose was used as an anionic exchanger, and the dialyzed sample was purified by ion exchange chromatography. Firstly, the packed exchange material within the column was washed with distilled water to remove the surplus reagents and buffer. The column was first equilibrated with 150 ml of 0.05 mol potassium phosphate buffer (pH 8.0) as counter ions and washed with the same buffer solution. The partially purified 5 ml cellulase concentrated with sucrose was loaded onto the surface of column (DEAE-cellulose). Then the linear gradient concentrations of NaCl ranged between 0.1–0.5 mol was used to elute the negatively charged bound proteins. The flow rate was 20 ml.h^-1^ and the elute was collected in 5 ml fraction. Total 20 fractions were collected, and their CMC activity was assessed. The fractions containing high specific activity were pooled and dialyzed against potassium phosphate buffer solution (pH 8.0) and further subjected to gel filtration.

#### Gel filtration chromatography

Gel filtration chromatographic technique was used for further purification of the cellulase enzyme. A volume of 15 ml of partially purified cellulase was applied on Sephadex G-100 column previously equilibrated with 0.1 mol potassium phosphate buffer (pH 8.0). The cellulase was eluted through the column matrix with a flow rate of 20 ml.h^-1^. Total 20 fractions were collected and analysed for the CMC activity as above. The fractions with higher cellulase activity were pooled and subjected for further analyses including Sodium Dodecyl Sulphate-Polyacrylamide Gel Electrophoresis (SDS-PAGE).

#### SDS-PAGE

The equal volumes of sample and buffer (5% 2-mercaptoethanol, 5% SDS in 0.5M Tris–HCl buffer; 0.1% bromophenol blue, pH 6.8 and 12% glycerol) were added and subjected to boiling at 100°C for 2 min. Then by using Omni PAGE Protein system (Cleaver Scientific), the samples were loaded onto SDS-PAGE (stacking gel: 5%, resolving gel: 12%). In each well approximately 40 μl of samples containing equal amount of proteins mixed with loading buffer in ratio 1:1 were loaded. The electrophoresis was run at room temperature with cooling pad inside buffer for 1 hr at 200 V having 10x SDS page running buffer. After the run, gel was washed with milliQ water and stained in Coomassie brilliant blue for 40 min. After staining gel was distained over night in solution containing (methanol, acetic acid and distilled water). The distaining was continued till the gel turned transparent and distinct blue bands were visible. The gel was photographed and analysed with a Gel Documentation system. Using SDS-PAGE method, the molecular mass of purified cellulase was determined with a standard molecular weight marker i.e., Page Ruler Plus Pertained Protein Ladder 10–250 kDa.

### Characterization of cellulase enzyme

#### pH optimization of cellulases produced by *B*. *subtilis* TD11

About 100 μl of cellulose enzyme was added to 900 μl of 1.0% CMC. The cellulase activity was measured after 20 hr of incubation at 50°C at various pH ranging 3.0 to 11.0 using different buffers i.e., pH 3.0 to pH 7.0: 50 m-mol citrate buffer; pH 6.0–9.0: 50 m-mol phosphate buffer; and pH 8–11:50 m-mol carbonate buffer. To measure the pH stability the relative cellulase activity was measure at different pH after every 01 hr. The assays were performed in triplicates and the results were expressed as mean ± standard error of means (SEM).

#### Temperature optimization of cellulases produced by *B*. *subtilis* TD11

About 100 μl of cellulase enzyme was added to approximately 900 μl of 1% CMC in 20 m-mol phosphate buffer (pH 7.0). The mixture was incubated at various temperatures ranging from 20–90°C for 20 hr and the cellulase activity was measured. Moreover, the cellulase stability at the different temperatures was assessed by analysing cellulase activity every hour for 20 hours. The assays were performed in triplicates and the results were expressed as mean ± SEM.

#### Substrate specificity of cellulases produced by *B*. *subtilis* TD11

To evaluate different substrate specificity of the purified cellulase, 1% CMC, hydroxyethyl cellulose (HEC), cotton fiber, xylan, vicel and filter paper were used as substrate. Briefly, 0.5 ml of 1% (w/v) of cellulosic substrates (CMC, HEC, Xylan, Avicel, Cotton fiber or filter paper) in 0.5 M citrate buffer (pH 4.0) was added to 0.1 ml of purified cellulase in a test-tube and mixed well. A strip of filter paper was used as substrate for the assays. The mixture was incubated at 50°C for 20 min. Then 3 ml of 3, 5-dinitrosalicyclic acid (DNS) solution was added to stop the reaction and the mixture was placed in hot water bath for 5 min. The mixture was then allowed to cool and 5 ml of distilled water was added. The activity of the enzyme was measured against each substrate using standard enzyme assays as given above.

### Extraction and purification of chitinase enzyme

#### Ion exchange chromatography

Using Ion Exchange chromatography, the dialyzed samples were purified. Firstly, the packed exchange material within the column was washed to remove the surplus reagents from the column. As counter ions, the column was equilibrated with 0.05 M potassium phosphate buffer (pH 6.0). About 10 ml of moderately purified samples were loaded to the surface of the column. Then columns were washed with the help of the buffer solution of potassium phosphate pH (6.0) to remove the unwanted material. After washing the column, the bound chitinase (negatively charged) was eluted using a linear gradient of sodium chloride concentrations ranging from 0.1 to 0.5 mol. The active fractions (0.2 and 0.3 mol) were allocated and dialyzed overnight against a potassium phosphate buffer solution (pH 6.0), followed by gel filtration.

#### Gel filtration chromatographic technique

A volume of 5 ml of partially pure chitinase was applied to gel-filtration of Sephadex G-100 column that had been pre-equilibrated with 0.1 mol potassium phosphate buffer (pH 6.0). Chitinase was eluted at a rate of 20 ml hr^-1^ via the column matrix. Fractions with chitinase activity were collected and subjected to electrophoresis (SDS-PAGE), enzyme activity assay and characterization [23].

#### SDS-PAGE

The purified enzyme was added to an equal volume of buffer solution (5% 2-mercaptoethanol, 12% glycerol, 0.1% bromophenol blue, and 5% SDS in 0.5 mol Tris–HCl buffer; pH 6.8), and then boiled for 3 min at 95°C. The resulted samples were then processed by using SDS-PAGE (stacking gel: 5%, resolving gel: 12%) and Protein system Omni PAGE (Cleaver Scientific). At room temperature electrophoresis was performed with a cooling pad inside buffer for 1 hr at 120 V having 10X SDS page running buffer. After the run, gel was washed with milliQ water and stained in Coomassie brilliant blue for 40 min. After staining gel was distained over night in solution containing (methanol, acetic acid and distilled water).The distaining was continued till the gel turned transparent and distinct blue bands were visible. The gel was photographed and analysed with a Gel Documentation system. Using SDS-PAGE method, the molecular mass of purified chitinase was determined with a standard molecular weight marker i.e. Page Ruler Plus Pertained Protein Ladder 10–250 kDa.

### Characterization of chitinase enzyme

#### Time optimization of chitinase produced by *B*. *subtilis* TD11

The optimum time of incubation for chitinase production was evaluated by incubating the bacterial culture flasks in a shaking incubator with150 rpm at 30°C for 10 days. The extracted culture filtrate was examined after every 24 hr for enzyme production and this process was repeated for a period of 10 days at 50°C [24]. The assays were performed in triplicates and the results were expressed as mean ± SEM.

#### Temperature optimization of chitinase produced by *B*. *subtilis* TD11

The culture flasks containing chitin media were incubated at different temperatures ranging from 20 to 40°C with shaking at 150 rpm for 24 hr at pH 7. Moreover, the effect of temperature on enzyme activity reaction was also evaluated. For this purpose, standard chitinase activity assays were performed as given above using the purified enzyme incubating for 20 hr at different temperatures ranging from 20 to 80°C with colloidal chitin as a substrate at pH 7 [23]. The chitinase activity was measured by taking absorption peak at 420 nm using a UV-Vis spectrophotometer. The assays were performed in triplicates and the results were expressed as mean ± SEM.

#### pH optimization of chitinase produced by *B*. *subtilis* TD11

The effect of initial pH of the chitinase activity was evaluated by adjusting the initial pH of the medium ranging from pH 4 to pH 9 at 30°C at 150 rpm for 24 hr [24]. The chitinase activity was measured by taking absorption peak at 420 nm using a UV-Vis spectrophotometer. The assays were performed in triplicates and the results were expressed as mean ± SEM.

#### Inhibitory effects of chitinase produced by *B*. *subtilis* TD11 on Fungal hyphae

Inhibitory effects of chitinase on different fungal hyphae (*Rhizoctonia*, *Colletotrichum*, *Aspergillus* and *Fusarium*) were measured using 24-well culture plates assays. Fungal hypal culture was prepared by inoculating 100ml potato dextrose broth medium followed by incubation at 28°C with shaking at 150 rpm for 3 days. Then 100 μl of fungal hyphal culture was added into the culture plate wells. The enzyme solutions in different volumes (50, 100, 200, and 400 μl) were added to the 100 μl of fungal hyphal culture. The positive and negative control wells contained no enzyme or fungal hyphae respectively. The plates were incubated for 2 days at 26°C and then inhibitory effects were assessed using ELISA Plate Reader at 405 nm [25].

## Results

### Lytic enzyme activity of *B*. *subtilis* TD11

**Fig 1:**
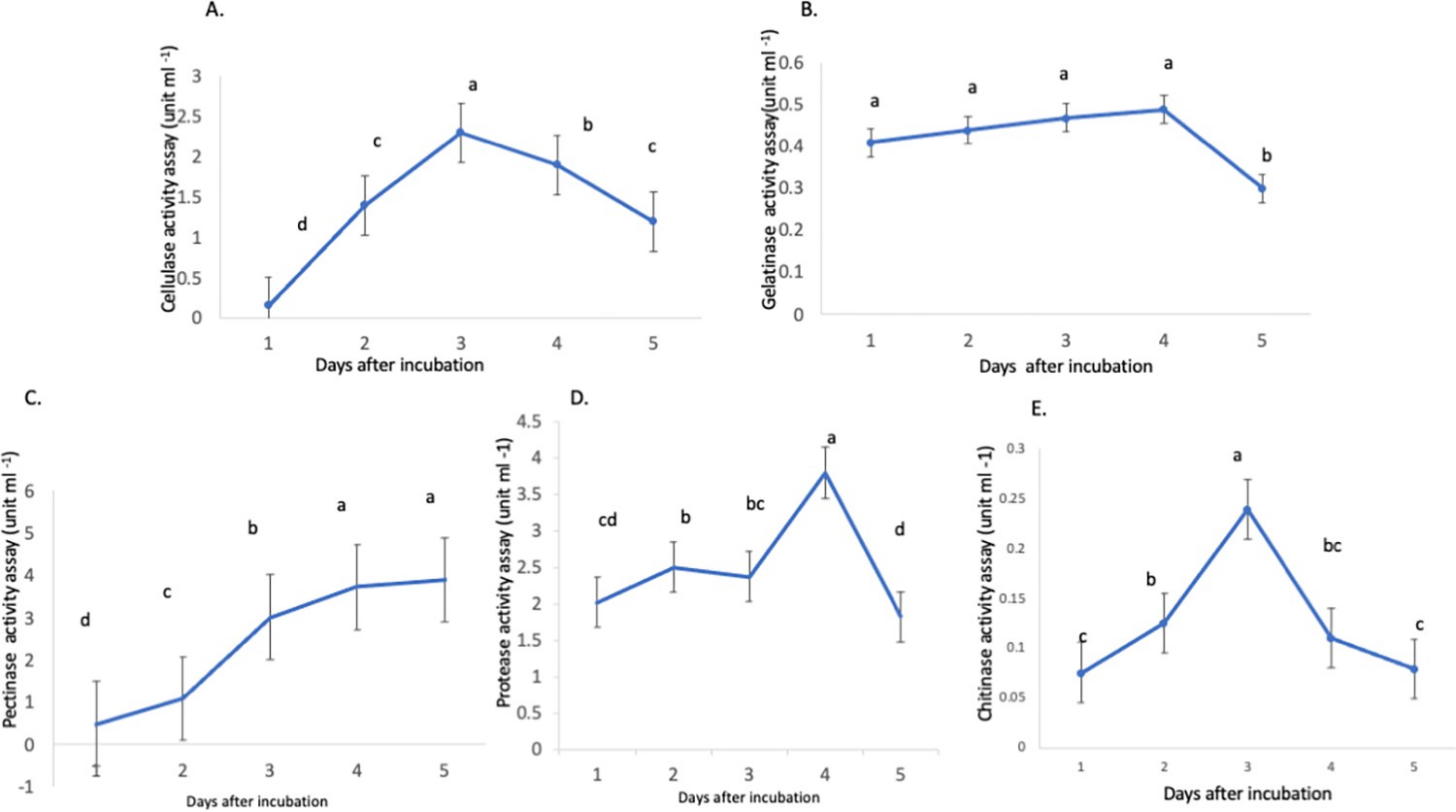
**(A)** Cellulase enzyme activity (unit-ml^-1^) of culture extract of *Bacillus subtilis* TD11 incubated for different durations using 1% carboxymethyl cellulose (CMC) as a substrate at pH 7 and temperature 50°C **(B)** Gelatinase enzyme activity (unit-ml^-1^) of culture extract of *B*. *subtilis* TD11 incubated for different durations using 0.5% gelatine as a substrate at pH 8 and temperature 37°C **(C)** Pectinase enzyme activity (unit-ml^-1^) of culture extract of *B*. *subtilis* TD11 incubated for different durations using 1% pectin as a substrate at pH 5 and temperature 45°C **(D)** Protease enzyme activity (unit-ml^-1^) of culture extract of *B*. *subtilis* TD11 incubated for different durations using skimmed milk as substrate at room temperature **(E)** Chitinase enzyme activity (unit-ml^-1^) of culture extract of *B*. *subtilis* TD11 incubated for different durations using 0.5% colloidal chitin as a substrate at pH 5.0 and temperature 37°C. The experiments were conducted in triplicate and the data were analysed using one-way ANOVA and the means were compared with the help of Fisher least significant difference (LSD) test. The different small letters on each data point for each enzyme activity represent significant differences.

Cellulase activity produced by the *Bacillus subtilis* TD11 significantly increased from Day 1 to Day 3 and then decreased with further incubation. The maximum activity was found at Day 3 (Fig 1A). The gelatinase activity significantly increased up till Day 4 and then decreased with incubation. Maximum activity was found at Day 4 showing (Fig 1B). In addition, the pectinase and protease activities similarly showed increase till Day 3 and then became exponential with incubation at Day 4 and Day 5 (Fig 1C and 1D). The chitinase activity also increased from Day 1 to Day 3 and then decreased upon incubation (Fig 1E). The enzymes overall showed significant activity at Day 3.

### Cellulase enzyme purification

In the current study, about 100 ml of culture filtrate was purified by ammonium sulphate (NH_4_)_2_SO_4_ using precipitation technique. About 15 ml of resultant precipitate was dialysed overnight at 4°C. The dialysate was passed through DEAE-cellulose column and further purified by gel filtration chromatography. The resultant activities and concentration of each step with purification fold and yield was determined (Table 1).

**Table 1:** The stepwise purification and characteristics of cellulase enzyme produced by *Bacillus subtilis* TD11.

Step	volume (ml)	Activity U-ml^-1^	Protein conc. (mg-ml^-1^)	Specific activity (U-mg^-1^)	Total activity (U)	Purification fold	Yield (%)
Crude enzyme	100	805	14	123	91350	1	100
Ammonium sulfate precipitate	15	602	8.7	140	56500	2	61.8
Ion exchange DEAE-cellulose	15	741	5.7	254	14240	2.5	15.5
Gel filtration Sephadex G-100	20	953	5.3	332	19124	3	20.93

**Fig 2:**
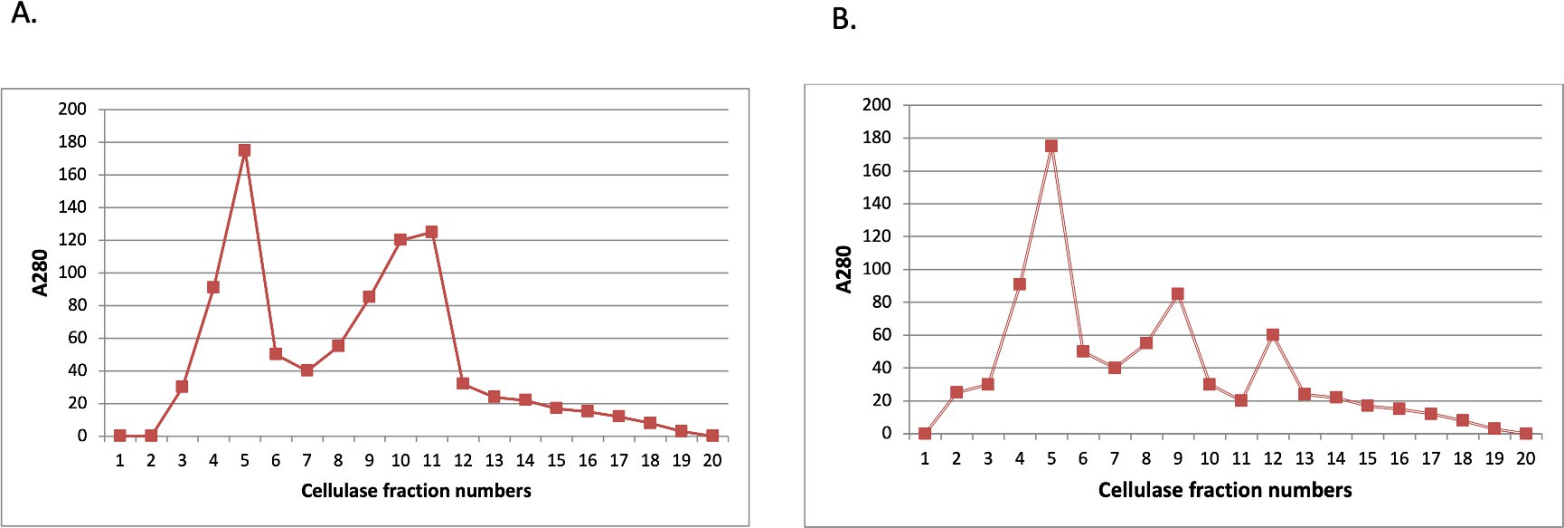
**(A)** Elution profile of DEAE-cellulose ion exchange chromatography of cellulase produced by *Bacillus subtilis* strain TD11 using linear gradient concentrations of NaCl ranged between 0.1–0.5 mol at a flow rate was 20 ml.h^-1^ and **(B)** elution profile of gel filtration on Sephadex G-100 of cellulase produced by *B*. *subtilis* strain TD11 with 0.1 mol potassium phosphate buffer (pH 8.0) at a flow rate of 20 ml.h^-1^.

The crude cellulase enzyme was first fractioned using DEAE-cellulose column. Out of the total 20 fractions collected, the fractions (4, 5, 9, 10 and 11) showed the highest peaks and were further proceeded for the estimation of proteins (Fig 2A). It was found that, specific activity increased from 140 to 254 U-mg^-1^ and the purification fold was 2.5 as shown in Table 1. On the other hand, the partially purified enzyme using DEAE-cellulose column was further purified on Sephadex G-100 column. The fractions 4, 5 and 9 showed the highest peak and the specific activity was found to increase from 254 to 322 U-mg^-1^ (Fig 2B) and the purification fold was found to be 3 (Table 1).

### SDS-PAGE profile of cellulase enzyme produced by *B*. *subtilis* TD11

**Fig 3:**
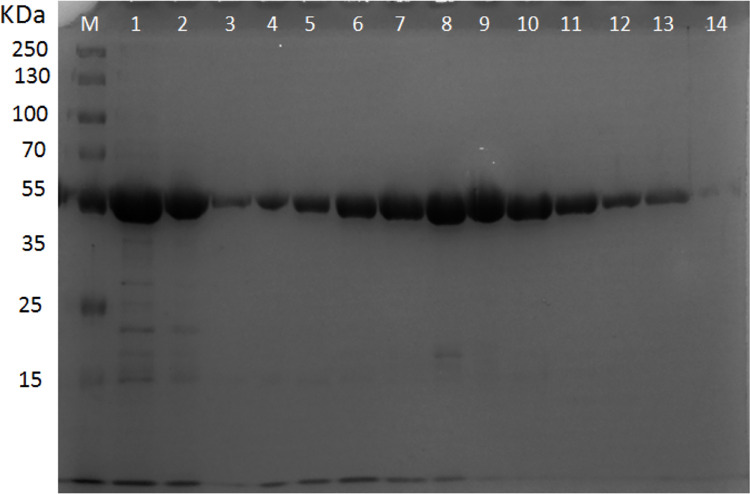
SDS-PAGE analysis of cellulase enzyme purified from *Bacillus subtilis* TD11. *Lane M* Shows Page Ruler Plus Pertained Protein Ladder (10–250 kDa). *Lane 1* shows crude enzyme, *Lane 2* shows fraction after DEAE-Cellulose ion exchange chromatography &*Lane 3–14* shows purified fractions of cellulase 45 kDa after gel filtration (Sephadex G-100).

Total of 12 fractions were collected during Sephadex gel filtration (Fraction number 3 to 14). The fractions were collected after intervals of 10 sec. It was found that maximum purification was obtained after 1 min (60 sec) at fraction number 8 followed by fraction number 9. The purification of cellulase enzyme was analyzed by SDS-PAGE. The analysis revealed that the molecular mass of purified cellulase obtained was 45 kDa using standard marker of molecular weight (Page Ruler Plus Pertained Protein Ladder 10–250 kDa) as shown in Fig 3.

### Effect of pH on the activity and stability of purified cellulase enzyme

**Fig 4:**
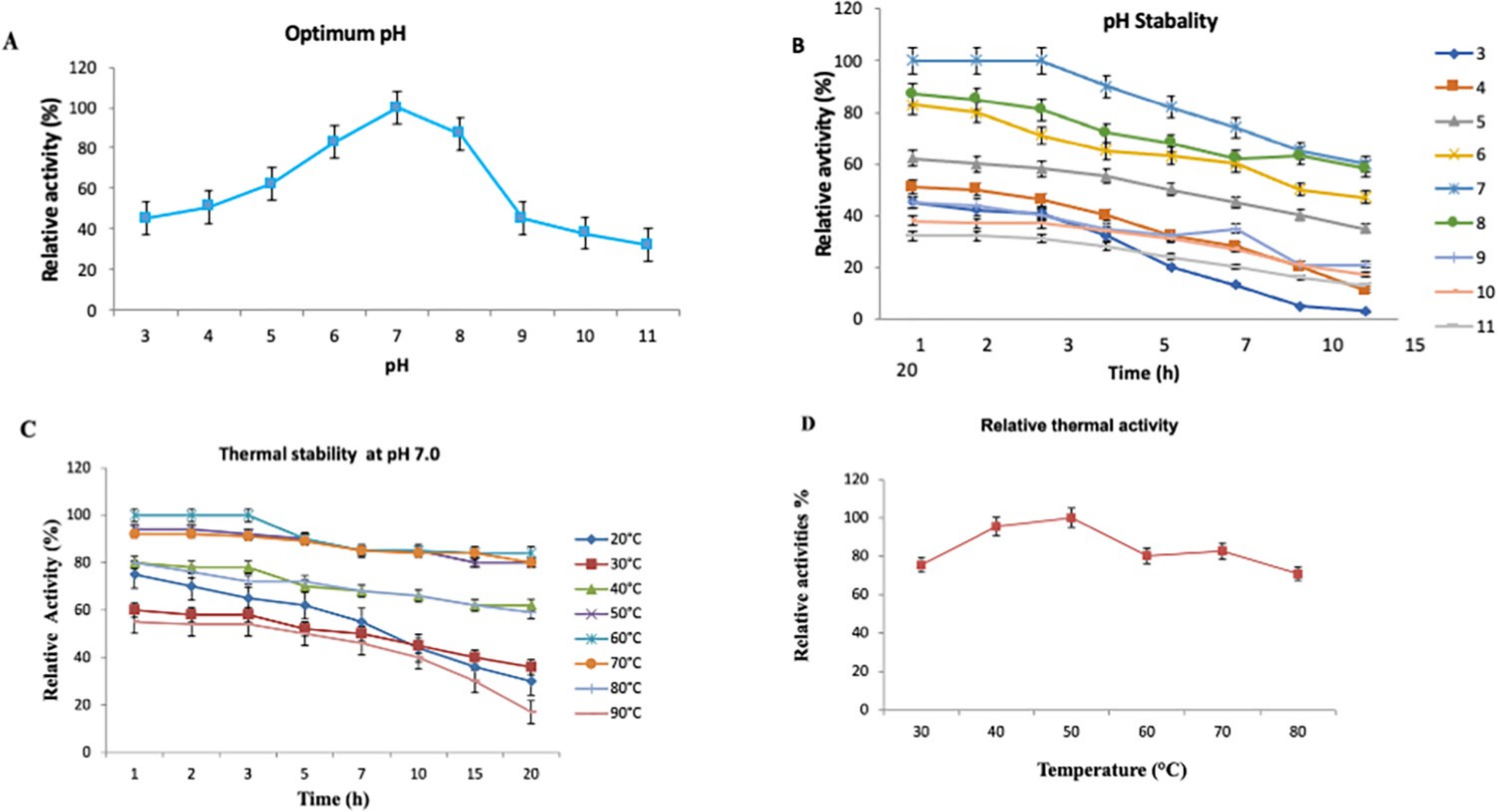
**(A)** Effect of pH on the activity of cellulase purified from *Bacillus subtilis* TD11 using carboxymethyl cellulose as substrate at various pH values ranging from 3.0–7.0 at 50°C **(B)** The pH stability of cellulase purified from *B*. *subtilis*TD11, the enzyme was incubated at 50°C for 1 h with 1.0% (w/v) CMC at pH 3.0 to 11.0 **(C)** Thermal activity of cellulase purified from *B*. *subtilis* TD11 at 20 to 90°C at pH 7 after 20hrs of incubation while stability of cellulase at 30 to 80°C at pH 7 after 1 hr of incubation and **(D)** Relative thermal activity of cellulase purified from *B*. *subtilis* TD11 at 30 to 80°C at pH 7 after 20hrs of incubation. The assays were performed in triplicates and the results were expressed as mean ± SEM.

Purified enzyme was analysed to check the effect of pH on the cellulase activity and its stability at different pH ranging from 3.0 to 11.0 (Fig 4A). It was found that the optimal pH for cellulase activity was 7.0 while more than 60% CMC activity was maintained at pH ranging from 4.0 to 9.0. More than 40% of the CMC activity was observed at pH ranging from 4.0 to 9.0 at 50°C after 20 hr of incubation (Fig 4B).

### Effect of temperature on activity of purified cellulase enzyme

The purified enzyme showed more than 80% of the CMC activity at 50–70°C after 20 hr incubation at pH 7.0 (Fig 4C). Furthermore, about 60% of the original CMC activity was conserved at 40–80°C. However, less than 40% of the original CMC activity was detected at 20, 30 and 90°C. Similarly, the temperature effect on the CMC activity of the purified cellulase enzyme was also analysed at temperatures ranging from 30–80°C at pH 7.0 and the residual activity in aliquots was assayed. After 1 hr of incubation, the purified cellulase produced maximum residual activity at 40–50°C. Relative activities for CMC at 30 to 80°C were 75.4, 95.5, 100, 80.2, 82.7 and 70.9% respectively as shown in Fig 4D.

### Determination of substrate specificity of purified cellulase enzyme

The highest activity was observed against CMC (100%) followed by the activity against HEC (57%). Less than 50% activity was observed when cotton fibre, crystalline substrates of avicel and xylan were used as substrates as shown in Table 2.

**Table 2:** Substrate specificity of the cellulase purified from *Bacillus subtilis* TD11 using 1% (w/v) of the substrates.

Substrates	Activity (%)
Carboxymethylcellulose	100
Hydroxyethyl cellulose	57
Filter paper	10
Avicel	42
Cotton	14
Xylan	0

### Characterization of chitinase enzyme

**Fig 5:**
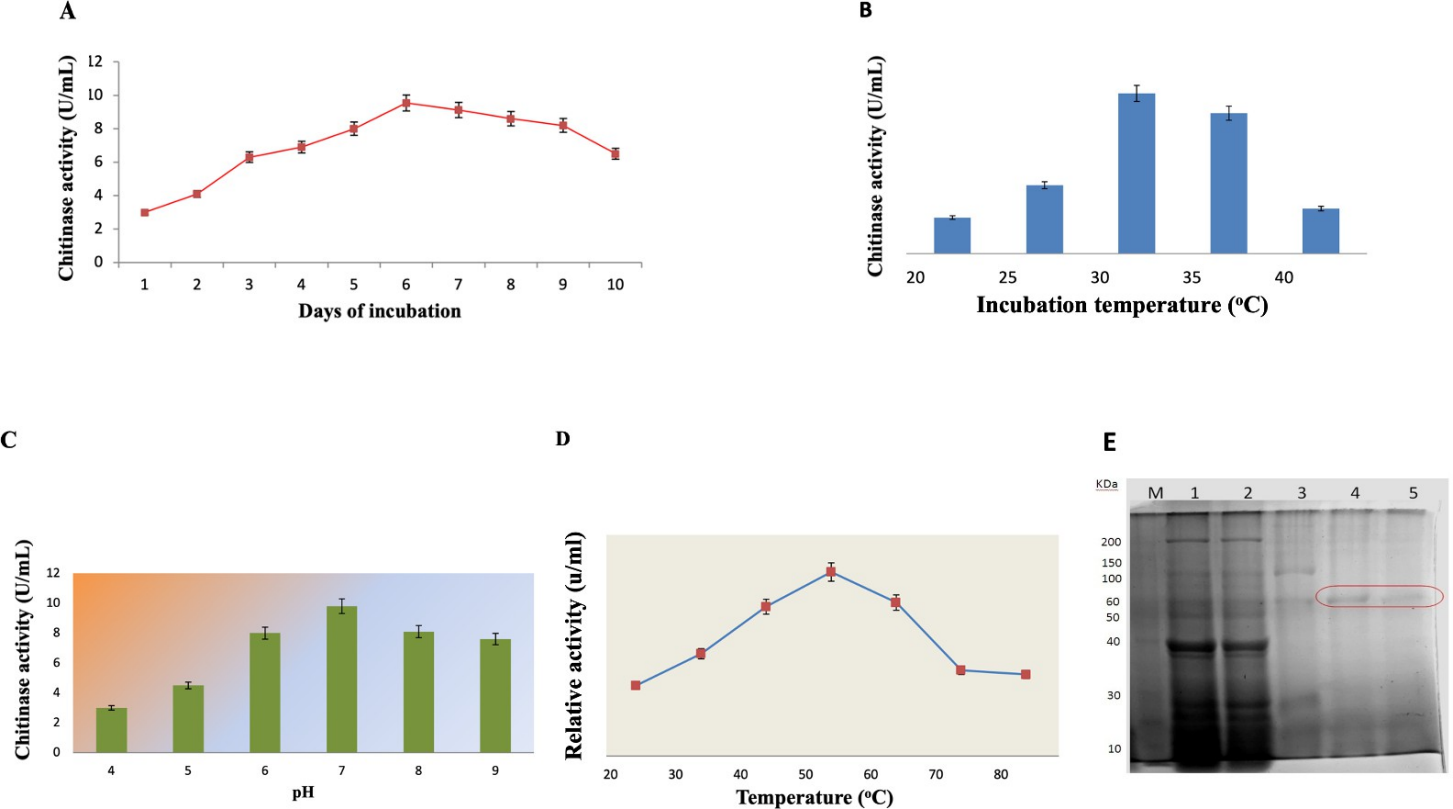
**(A)** Production of chitinase by *Bacillus subtilis* TD11 at different intervals of incubation (days after incubation) at pH 7 and 30°C, **(B)** Effect of temperature on chitinase production at pH 7 after 24 hr of incubation, **(C)** Effect of initial pH of the fermentation media on chitinase production after 24 hr of incubation, **(D)** Effect of different temperature on chitinase activity after 20 hr of incubation at pH 7 (E) SDS-PAGE analysis of Chitinase Enzyme. *Lane M* Show Bio basic wide range molecular weight Marker (Catalog# BSM 0661), *Lane 1* shows crude enzymes, *Lane 2* shows fraction after precipitation by 80% SAS, *Lane 3* Shows enzyme column purified fraction and *Lane 4 & 5* shows the purified fraction of Chitinase approximately 55 kDa after gel filtration chromatography (Sephadex G-100). The assays were performed in triplicates and the results were expressed as mean ± SEM.

The maximum chitinase yield was found after incubation at Day 6 (9.54 U-ml^-1^), and then the production declined with further incubation (Fig 5A). Change in temperature of incubation (20–40°C) had a significant effect on the chitinase activity produced by *B*. *subtilis* TD11. The maximum production was found to be 8.9 and 7.8 U-ml^-1^ at 30°C and 35°C respectively (Fig 5B).The optimum chitinase production was found at pH 7 with maximum activity of 9.8 U-ml^-1^. A minimum chitinase activity of 3.0 U-ml^-1^ was observed at pH 4 (Fig 5C). The optimum temperature for chitinase activity was found to be 50°C with an activity of 13.31 U-ml^-1^. Any deviation from this temperature led to a gradual decrease in the enzyme activity. The relative activity of chitinase was 80% at 40 and 60°C (Fig 5D).

### Purification of chitinase

Approximately 55 kDa molecular mass of purified chitinase was obtained in comparison with a standard molecular weight marker (Bio basic wide range Molecular weight Marker Catalog# BSM 0661) as shown in Fig 5E.

### Inhibitory effects of chitinase produced by *B*. *subtilis* TD11 on Fungal hyphae

Purified chitinase enzyme was subjected to a well culture plate assay with different concentrations against different fungal phytopathogens. It was observed that the chitinase enzyme inhibited the growth fungal pathogens including *Fusarium oxysporum*, *Rhizoctonia solani*, *Colletotrichum* sp. and *Aspergillus* sp. by hyphal degradation measured by the respective optical densities at 405 nm (Table 3).

**Table 3:** The antifungal activity of purified chitinase produced by *Bacillus subtilis* TD11 using well diffusion assays against the supernatant containing metabolites of different plant pathogenic fungi including *Fusarium oxysporum*, *Rhizoctonia solani*, *Colletotrichum* Sp. and *Aspergillus* Sp. Optical density was recorded at 405 nm using LEDTECH 96 ELISA Plate Reader.

Name of Fungus	Fungus before treatment (100μl)	Different combinations of Purified Chitinase + Fungus in microliter				Purified Chitinase without Fungus (100 μl)
		50 μl +100 μl	100 μl + 100 μl	200 μl + 100 μl	400 μl + 100 μl	
*Fusarium oxysporum*	0.225	0.195	0.142	0.115	0.095	0.064
*Rhizoctonia solani*	0.254	0.189	0.144	0.117	0.098	0.057
*Colletotrichum* Sp.	0.241	0.196	0.148	0.116	0.091	0.048
*Aspergillus* Sp.	0.253	0.193	0.137	0.113	0.096	0.054

## Discussion

In the current study, a recently reported potent antagonistic bacterial strain *B*. *subtilis* TD11 [19] was assessed for its ability to produce lytic enzymes of industrial and biotechnological significance under different culture conditions. Malik et al. [19] previously identified the strain of *B*. *subtilis* TD11 as a promising putative biocontrol agent that significantly inhibited fungal diseases of tomato caused by ascomycetous and basidiomycetous fungi (*Fusarium oxysporum* and *Rhizoctonia solani* respectively). In the present study, this strain showed significant lytic activities including cellulase, gelatinase, protease, chitinase and pectinase. These results demonstrated the strain *B*. *subtilis* TD11 as a good producer of versatile lytic enzymes. Here the cellulase and chitinase enzymes produced by *B*. *subtilis* TD11 were purified using standard extraction, and purification techniques. The purified enzymes were then optimized in different culture conditions such as pH, temperature and substrate specificity. The inhibitory effects of the purified chitinase on different chitin containing hyphae of fungi belonging to genera of *Rhizoctonia*, *Colletotrichum*, *Aspergillus and Fusarium* were also determined.

In this study, the cellulase produced by *B*. *subtilis* TD11 had a molecular weight of 45 kDa and it was based on their electrophoretic mobility with respect to reference proteins. Moreover, the purified cellulase enzyme demonstrated a 20-fold increase in activity with a 15% recovery yield. According to the previous reports, the molecular mass of cellulase varies not only among different types of organisms but also among different strains of the same species of bacteria. Yin et al. [26] purified cellulase from *B*. *subtilis* YJ1 with a molecular weight of 32.5 kDa that showed 289 fold increase in activity and 9.7% recovery yield upon purification. In another study, a thermostable cellulase purified from *B*. *subtilis* DR had a molecular weight of 55 kDa [27]. Yet another study reported purification of cellulase from *B*. *subtilis* LFS3 with molecular weight of 185 kDa securing 15% recovery yield upon purification [28]. Asha et al. [29] purified cellulase with a molecular weight of 51.4 kDa. Pramanik et al. [30] reported production of cellulase with 44.7 kDa by *B*. *pseudomycoides*. Islam and Roy [31] purified cellulase with molecular weight of 67 kDa from another species of bacteria (*Paenibacillus* sp.). The variability in sizes of cellulase protein produced by bacteria also shows an enormous diversity in genes responsible for its production and accordingly their properties also vary.

The environmental factors have impact on the enzyme activities especially the pH and temperature of the media. Our results showed that the optimum pH for the cellulase activity was 7.0 and the activity was more than 60% of the purified cellulase between pH 4 and 9.0. Furthermore, it was observed that the purified cellulase was stable at pH 6.5–7.5. In literature, however, a variability at intraspecific level exists in optimal pH values for cellulases produced by different strains of *B*. *subtilis*. Yin et al. [26] purified cellulase from *B*. *subtilis* YJ1 with an optimal pH of 6.0. Yet another strain of *B*. *subtilis* LFS3 produced an acidophilic cellulase with an optimum at pH 4 [28]. *Bacillus subtilis* SU40 produced an alkalophilic cellulase with an optimum pH of 8.0 [29]. Previous studies also suggested generally the optimal pH for cellulases purified from *Bacillus* strains [32] being stable at pH 5.0–6.5, that from *Aspergillus niger* [33] at 6.0–7, and that from *Lysobacter sp*. at 5.0–7.0. Each enzyme has an optimum pH range and changing the pH beyond this range would slow down enzyme activity and extreme pH values may often cause enzymes to denature. However, increasing enzyme concentration may speed up the reaction even at higher pH range, if there is substrate available to bind to.

In the present study, the optimum temperature for the purified cellulase for CMCase activity were evaluated and it was observed that the purified cellulase showed more than 80% of the CMC activity at 50–70°C after 20 hr incubation at pH 7.0. Furthermore, around 60% of the original CMC activity was conserved at 40–80°C whereas, less than 40% of the original CMC activity was detected at different temperature ranges i.e., 20, 30 and 90°C. Our findings are consistent with those of Azadian et al. [12] who also reported thermophilic CMCase with optimum temperature of 70°C. Similarly, optimum temperature for the cellulase purified from *B*. *subtilis* YJ1 was reported as 50–60°C [26]. The cellulase purified from *B*. *subtilis* LFS3 showed optimum activity at 60°C [28]. The optimum temperature for cellulase purified from *B*. *subtilis* SU40 was 45°C [29]. Generally cellulases produced by *Bacillus* species are thermophilic although there are some examples which show more activity at temperatures below 50°C.

In the present study, the purified cellulase was more active against CMC and was unable to hydrolyse Xylan. Most of the cellulases produced by *Bacillus* strains are capable of hydrolysing CMC [28, 29]. Cellulase purified from *B*. *amyloliquefaciens* SS35 showed a minimal activity against Xylan [34]. *Bacillus subtilis* YJ1 produced a cellulase that showed 14% of the relative activity against xylan [26]. However, the cellulases purified from *B*. *subtilis* LFS3 and *B*. *subtilis* SU40 could degrade Xylan [28, 29]. Further in our case, more than 40% of the CMCase activity was observed against HEC and Avicel while the activity decreased to 10 and 14% in case of filter paper and cotton respectively. *Bacillus amyloliquefaciens* SS35 showed around 2 U-mg^-1^ of specific activity (50% of relative activity) against HEC [34]. Deka et al. [35] also reported a cellulase purified from *B*. *subtilis* AS3 that showed 2.2 U-mg^-1^ of specific activity against HEC at 45°C after 10 min. Cellulase activity of 34% against avicel was reported by Yin et al. [26]. Asha et al. [29] also reported cellulases purified from *B*. *subtilis* that degraded avicel. However, no activiy or minimal activity was reported against avicel in other cases [34, 35]. Yin et al. [26] and Deka et al. [35] also reported minimal activity against filter paper and cotton respectively by different cellulases purified from different strains of *B*. *subtilis*.

In the present study, it was found that the chitinase produced by *B*. *subtilis* TD11 had a molecular weight of 55 kDa. Different molecular weights of chitinases had been reported in literature. For instances the chitinase purified from *B*. *subtilis* NPU 001 had a molecular weight of 31 kDa [36]. Similarly, Songsiriritthigul et al. [37] reported molecular weight of 35–55 kDa for chitinases produced by *Bacillus* spp. Mehmood et al. [38] also reported chitinases with molecular weight of 48 kDa produced by *Bacillus cereus* YQ308. Hence the molecular weight of enzymes as compared with the literature are highly variable and depend on the strain that produces them. Furthermore, in the present study the maximum chitinase production was found at pH 7 with the activity of 9.8 U-ml^-1^ while, the minimum chitinase activity of 3.0 U-ml^-1^ was observed at pH 4. This shows the chitinase produced by *B*. *subtilis* TD11 is a neutral enzyme. Yan et al. [39] also reported an optimum pH of 7 for chitinase produced by *B*. *subtilis* SL-13 being stable at pH 5–9. Kim et al. [36] also reported similar pH range (5–9) for the stability of chitinase produced by *Serratia* sp. On the other hand, *B*. *subtilis* TV-125 produced chitinase with optimum pH of 4, however, that could retain its 71% activity even at pH 11 [40].

There is no doubt temperature has an impact on various biological processes, therefore the growth of bacteria and the production of enzymes are influenced by the incubation temperature alteration. The maximum production of chitinase by *B*. *subtilis* TD11 was found at temperature 30–35°C in this study and production decreased when temperature was increased or decreased. In addition to that, the purified chitinase enzyme showed maximum activity of 13 U ml^-1^ at 50°C. The present study indicated that the production of enzyme was directly dependent on temperature and increase in temperature from the optimum range probably led to the enzyme deactivation. Kim et al. [36] also reported similar temperature range 30–35°C for chitinase enzyme produced by *Serratia* sp. while, Songsiriritthigul et al. [37] reported a higher temperature range (35–40°C) for chitinase enzyme produced by *B*. *licheniformis*. Moreover, there are reports of chitinases purified from different strains of *B*. *subtilis* showing their optimum activity at 50°C [39, 40].

The fungi contain chitin in their cell walls which is α- and β- linked glucan, and glycoproteins pigments. The chitinases can, therefore, play significant role in controlling fungi and produce of carbohydrates and receptors that may perform key events during interaction with the host. In the current research study, antifungal activity was demonstrated in well culture assay by the chitinolytic enzyme purified from the culture fluid of *B*. *subtilus* TD11. There was a gradual increase in the inhibition with the increase in the quantity of the enzyme. Chitinase enzyme was effective against all the fungi used in this assay. These results supported the use of *B*. *subtilis* in the protection of agricultural crops against fungal pathogens. Joo [41] also reported antagonistic properties of chitinolytic enzymes purified from *Streptomyces halstedii* towards fungal phytopathogens. Wang et al. [42] purified chitinases from *B*. *amyloliquefaciens* V656 that were antifungal. On the other hand, chitinolytic enzymes produced by *B*. *subtilis* TD11 displayed catalytic activity to convert complex chitin compounds into useful by-products as a result fungal strains lost their basic structure and hence become died.

## Conclusions

It was concluded that the cellulase and chitinase enzymes produced by *B*. *subtilis* TD11 were neutral as well as thermophilic in nature. The molecular masses of cellulase and chitinase produced by the bacterial strain was 45 and 55 kDa respectively. The cellulase was most active against CMC substrate. The chitinase inhibited the growth of hyphae having chitin in their cell walls.
